# Detection of Human GPCR Activity in Drosophila S2 Cells Using the Tango System

**DOI:** 10.3390/ijms26010202

**Published:** 2024-12-29

**Authors:** Emil Salim, Aki Hori, Kohei Matsubara, Toshiyuki Takano-Shimizu, Andre Rizky Pratomo, Marianne Marianne, Armia Syahputra, Dadang Irfan Husori, Asuka Inoue, Maryam Aisyah Abdullah, Nur Farisya Shamsudin, Kamal Rullah, Takayuki Kuraishi

**Affiliations:** 1 Faculty of Pharmacy, Institute of Medical, Pharmaceutical and Health Sciences, Kanazawa University, Osaka 920-1192, Japan; horiaki1102@staff.kanazawa-u.ac.jp (A.H.); andrer.pratomo@gmail.com (A.R.P.); 2 Department of Pharmacology and Clinical/Community Pharmacy, Faculty of Pharmacy, Universitas Sumatera Utara, Medan 20155, Indonesia; marianne80@usu.ac.id (M.M.); dadang@usu.ac.id (D.I.H.); 3 KYOTO Drosophila Stock Center, Kyoto Institute of Technology, Saga Ippongi-cho 1, Ukyo-ku, Kyoto 616-8354, Japan; dgrc-tec@kit.ac.jp (K.M.); fruitfly@kit.ac.jp (T.T.-S.); 4 Departement of Periodontology, Faculty of Dentistry, Universitas Sumatera Utara, Medan 20155, Indonesia; armia.syahputra@usu.ac.id; 5 Graduate School of Pharmaceutical Sciences, Tohoku University, 6-3, Aoba, Aramaki, Aoba-ku, Sendai 980-8578, Japan; iaska@tohoku.ac.jp; 6 Graduate School of Pharmaceutical Sciences, Kyoto University, 46-29 Yoshida-Shimo-Adachi-cho, Sakyo-ku, Kyoto 606-8501, Japan; 7 Department of Pharmaceutical Chemistry, Kulliyyah of Pharmacy, International Islamic University Malaysia, Bandar Indera Mahkota, Kuantan 25200, Pahang, Malaysia; maryamaisyah9221@gmail.com (M.A.A.); farisya@iium.edu.my (N.F.S.); kamalrullah@iium.edu.my (K.R.)

**Keywords:** human GPCRs, *Drosophila* S2 cells, Tango system, human DRD4, dopamine

## Abstract

G protein-coupled receptors (GPCRs) are essential cell surface proteins involved in transducing extracellular signals into intracellular responses, regulating various physiological processes. This study validated the use of the Tango assay, a sensitive method for detecting GPCR activation, in *Drosophila* Schneider 2 (S2) cells, focusing on the human Dopamine Receptor D4 (DRD4). Plasmids encoding the LexA-tagged human DRD4 receptor and a luciferase reporter were co-transfected into *Drosophila* S2 cells and stimulated with dopamine. Receptor activation was measured by quantifying the luciferase activity. The system showed high specificity for dopamine, with no activation in response to octopamine, a non-ligand for DRD4. Furthermore, the system effectively detects activation by a novel compound. These results demonstrate that *Drosophila* S2 cells, coupled with the Tango assay, provide a viable model for studying human GPCR function and ligand specificity. This system enables the rapid screening of potential GPCR ligands in a cost-effective cellular model.

## 1. Introduction

G protein-coupled receptors (GPCRs) represent the most extensive family of cell membrane proteins, with over 800 genes coding for them in the human genome. These receptors are essential for controlling numerous physiological functions [1,2]. Ligand binding induces a conformational change in GPCRs, which in turn activates the associated G protein, leading to the regulation of various downstream signaling pathways [3,4,5].

Identification of ligands for G protein-coupled receptors (GPCRs) is critical for deciphering cellular responses to environmental stimuli. Ligands interacting with GPCRs can function as agonists, which activate the receptor, or antagonists, which inhibit its activity, resulting in diverse cellular effects [6,7]. This understanding is particularly important in the field of drug discovery because GPCRs represent one of the most extensively targeted classes of receptors in pharmacology [8]. Notably, more than one-third of all approved pharmaceuticals exert their effects by modulating GPCR activity, encompassing treatments for conditions, such as cardiovascular diseases, mental health disorders, and cancer [9]. Consequently, the identification and characterization of GPCR ligands remain pivotal in the development of more selective and efficacious therapeutic agents with reduced side effects [10].

Traditional assays for detecting GPCR activity, such as monitoring cAMP production and calcium mobilization, have limited applicability across different GPCRs. These assays primarily capture second messenger activities associated with specific G proteins, such as Gs, Gi, or Gq, leaving pathways involving G12/13 poorly characterized [11]. The Tango assay addresses these limitations by offering broad applicability across different GPCRs by exploiting ligand-mediated arrestin binding; virtually all GPCRs associate with arrestin upon activation, enabling the Tango assay to measure activation across a wide range of GPCRs, including those coupled with all known G protein classes [12]. This assay was designed to monitor GPCR activation by utilizing β-arrestin recruitment as a signaling mechanism and converts transient GPCR-β-arrestin interactions into a stable and quantifiable reporter gene signal, such as luciferase. Specifically, the assay involves fusing β-arrestin to a protease that cleaves a membrane-tethered transcription factor upon receptor activation. This released transcription factor then moves to the nucleus and activates a reporter gene, allowing for a sensitive and long-lasting readout of receptor activation. The Tango assay demonstrates high selectivity by being immune to signaling from endogenous receptors, setting it apart from assays based on second messenger accumulation, which are susceptible to background interference. This feature makes the Tango assay a general but selective tool for all GPCRs, regardless of their G protein type [12]. Furthermore, the Tango assay is particularly advantageous when G protein activation of second messengers is weak, or when the downstream G protein identity is unknown, making it an ideal choice for studying complex GPCR signaling pathways [12]. These qualities make the Tango assay a robust and versatile method for GPCR research.

*Drosophila* Schneider 2 (S2) cells have become increasingly favored for the investigation of various cellular mechanisms [13]. Initially derived from trypsinized late-stage embryos of Oregon-R *Drosophila melanogaster*, these cells retain characteristics akin to macrophages [14]. Unlike mammalian cells, S2 cells can be cultured at room temperature in simple media without requiring CO₂ supplementation, which significantly reduces the overall cost of maintenance [15]. These cells also demonstrate good transfection efficiency and expression levels [13,15,16]. Moreover, S2 cells are amenable to suspension culture and can be expanded using bioreactors, making them ideal for large-scale experiments [17,18]. Importantly, *Drosophila* cells are expected to lack endogenous ligands for human GPCRs, potentially resulting in a lower background noise and improved signal specificity.

In the present study, we employed the Tango assay within *Drosophila* S2 cells to detect the activation of the human GPCR, dopamine Receptor D4 (DRD4) by its ligand, dopamine. By expressing the human DRD4 receptor in S2 cells and utilizing the Tango system, we investigated whether *Drosophila* cells could be a suitable model for investigating human GPCR function and ligand specificity. We also assessed the potential of *Drosophila* S2 cells as a platform for screening novel compounds targeting GPCRs [19]. To achieve our objectives, we constructed plasmids encoding the LexA-tagged DRD4 receptor (UAS-DRD4-hTango) and luciferase reporter (pJFRC-8XlexAop-NanoLuc). These plasmids, along with the pMT-GAL4 plasmid, were co-transfected into *Drosophila* S2 cells and subsequently stimulated with dopamine, a natural ligand for DRD4. Receptor activation was detected using the Tango system to quantify the luciferase activity. To validate the system specificity, we applied cells with octopamine, a non-ligand for DRD4, and subsequently evaluated the system’s capacity to detect DRD4 activation by a novel compound. Finally, we tested the functionality of the system in vivo.

## 2. Results

### 2.1. Design of a Human DRD4-Tango System

**Figure 1:**
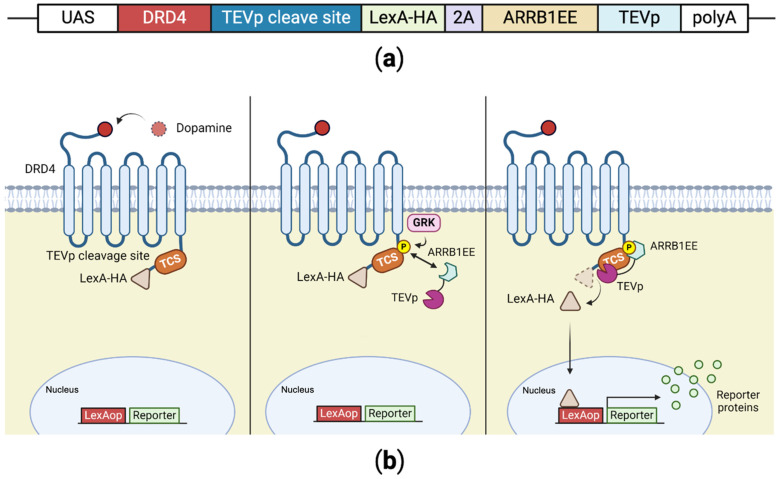
Schematic Diagram of the human DRD4 Tango System. (**a**) Design of the human DRD4 Tango transgene. (**b**) Illustration of the DRD4-hTango mechanism (Created with BioRender.com).

We sought to develop a genetically engineered tool capable of reporting the activation of human DRD4 expressed in *Drosophila* S2 cells. To achieve this, we adapted the Tango system [12] for use in *Drosophila* cells. This system converts a transient ligand/receptor interaction into a durable, quantifiable readout of reporter gene expression. The reporter gene is activated through a specialized, synthetic signaling pathway, which involves a bacterial transcription factor (lexA) that is covalently linked to the exogenous human DRD4 receptor, expressed in the target *Drosophila* S2 cells via a specific tobacco etch virus (TEV) protease-sensitive cleavage site (TCS) (Figure 1a). Upon ligand binding, the G protein coupled receptor kinase (GRK) phosphorylates threonine and serine residues in the DRD4 receptor and promotes arrestin (ARRB1EE) binding to the site. Subsequently, the transcription factor is cleaved from the DRD4 receptor by an arrestin-TEV protease fusion protein and then translocates to the nucleus, where it activates a lexAop-driven reporter gene (Figure 1b). Although this system was initially developed for detecting receptor activation in cultured mammalian cell lines [12], its effectiveness in detecting human GPCR activation in *Drosophila* S2 cells remained unexplored.

### 2.2. Validation of the Human DRD4-Tango System In Vitro

**Figure 2:**
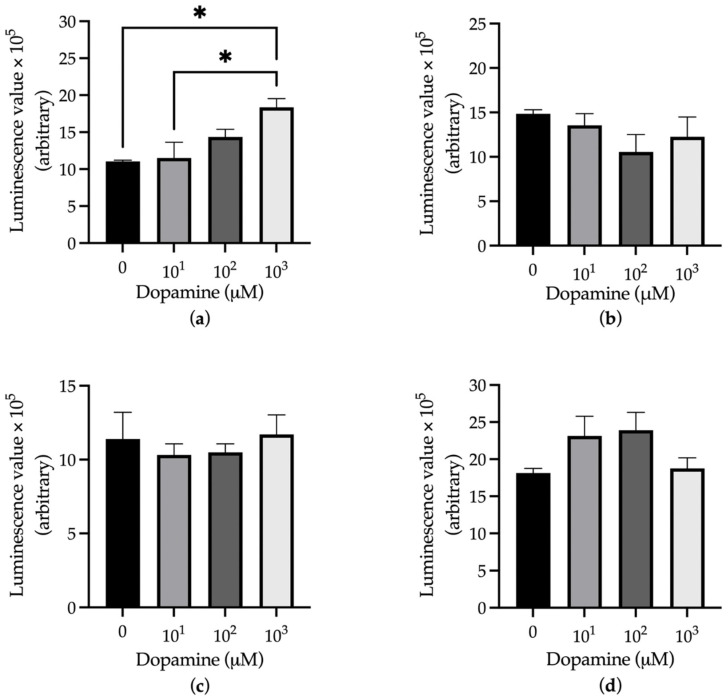
DRD4-Tango Reporter Analysis In vitro. DRD4-Tango reporter (luciferase) activity in response to dopamine in *Drosophila* S2 cells co-transfected with pMT-GAL4, UAS-DRD4-Tango, and LexAop-Luc following (**a**) 1 h, (**b**) 2 h, (**c**) 3 h, and (**d**) 4 h of incubation. Error bars represent the standard error of the mean (SEM). Asterisks indicate statistically significant differences (*p* < 0.05, ANOVA followed by Tukey test, *n* = 3).

To evaluate whether DRD4-Tango could report cellular activation by dopamine, we co-expressed human DRD4-Tango with a lexAop-luciferase reporter in *Drosophila* S2 cells. Upon treatment with dopamine, we observed an increase in reporter gene expression within 1 h of incubation (Figure 2a). However, no significant changes in reporter gene expression were detected after 2, 3, and 16 h of incubation (Figure 2b–d). These findings demonstrate that (1) the combination of human DRD4 and arrestin functions effectively to establish a viable Tango system, and (2) human DRD4-Tango successfully activates reporter gene expression in response to dopamine receptor ligands.

### 2.3. Selectivity Testing of the Human DRD4-Tango System In Vitro

**Figure 3:**
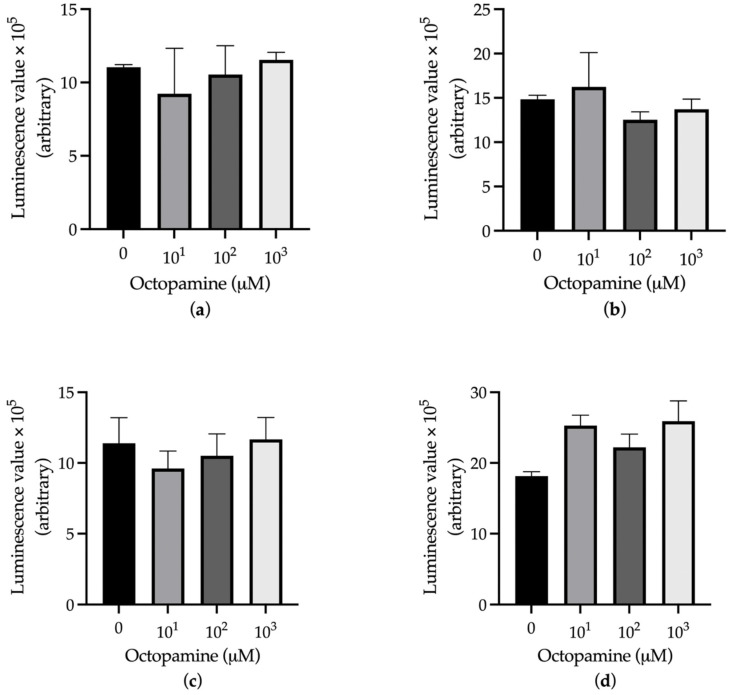
Specificity of the DRD4-Tango System. DRD4-hTango reporter (luciferase) activity in response to octopamine in *Drosophila* S2 cells co-transfected with pMT-GAL4, UAS-DRD4-Tango, and LexAop-Luc following (**a**) 1 h, (**b**) 2 h, (**c**) 3 h, and (**d**) 4 h of incubation. Error bars represent the standard error of the mean (SEM), *n* = 3.

To assess the selectivity of the human DRD4-Tango system for its native ligand dopamine, we tested its response to octopamine, a structurally similar non-DRD4 ligand. Octopamine, a biogenic phenylethanolamine, is well-characterized as a neurotransmitter and hormone in invertebrates [20]. Despite its structural resemblance to dopamine, treatment with octopamine at various time points (1, 2, 3, and 16 h) failed to induce a significant increase in luciferase activity, as shown in (Figure 3a–d). These findings unequivocally demonstrate the high ligand selectivity of the DRD4-Tango system, as it successfully differentiates dopamine from a structurally similar biogenic amine. This result is pivotal, as it underscores the reliability of the Tango system in detecting DRD4-specific activation while avoiding cross-reactivity with non-specific ligands. The observed selectivity is critical for ensuring robust signal specificity, an essential feature for GPCR-targeted drug discovery platforms.

### 2.4. Testing of a Novel Compound Using the Human DRD4-Tango System In Vitro

**Figure 4:**
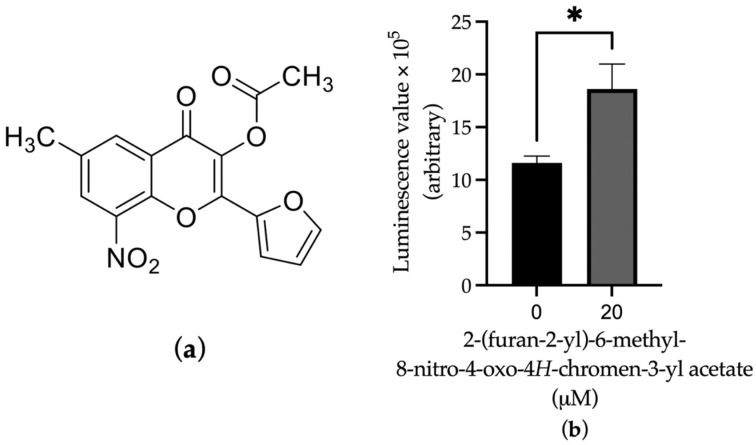
Activation of DRD4 by a Novel Compound. (**a**) Chemical structure of 2-(furan-2-yl)-6-methyl-8-nitro-4-oxo-4*H*-chromen-3-yl acetate (**b**) DRD4-hTango reporter (luciferase) activity in response to 2-(furan-2-yl)-6-methyl-8-nitro-4-oxo-4*H*-chromen-3-yl acetate in *Drosophila* S2 cells co-transfected with pMT-GAL4, UAS-DRD4-Tango, and LexAop-Luc following 1 h of incubation. Error bars represent the standard error of the mean (SEM). Asterisks represent statistically significant differences (*p* < 0.05, *t*-test, *n* = 3).

We employed a series of novel synthetic compounds based on the chromone scaffold to assess the performance of the human DRD4-Tango system. These compounds, developed by our research group, include several derivatives with promising activity profiles [21]. Among the tested compounds, 2-(furan-2-yl)-6-methyl-8-nitro-4-oxo-4*H*-chromen-3-yl acetate (Figure 4a) exhibited the most significant activity. The compound significantly enhanced luciferase activity, providing compelling evidence for successful activation of the DRD4 receptor (Figure 4b). This result aligns with our newly developed model for targeting DRD4, which accurately predicts the ligand’s binding affinity and receptor activation potential. The success of the model in identifying this novel DRD4 agonist demonstrates its utility in streamlining the drug discovery process for dopamine receptor-targeted therapeutics.

We further evaluated the utility of the DRD4-Tango system by testing a library of novel synthetic compounds developed by our research group, all derived from the chromone scaffold [21]. Among these, 2-(furan-2-yl)-6-methyl-8-nitro-4-oxo-4*H*-chromen-3-yl acetate (Figure 4a) exhibited the most significant activity. Treatment with this compound significantly enhanced luciferase activity, indicating robust activation of the DRD4 receptor (Figure 4b). This result provides compelling evidence that the novel compound functions as a potent agonist of DRD4. This result aligns with our newly developed model for targeting DRD4, which accurately predicts the ligand’s binding affinity and receptor activation potential. The successful identification of this DRD4 agonist highlights the utility of the DRD4-Tango system as a powerful tool for drug discovery for dopamine receptor-targeted therapeutics.

### 2.5. Validation of a Human DRD4-Tango System In Vivo

**Figure 5:**
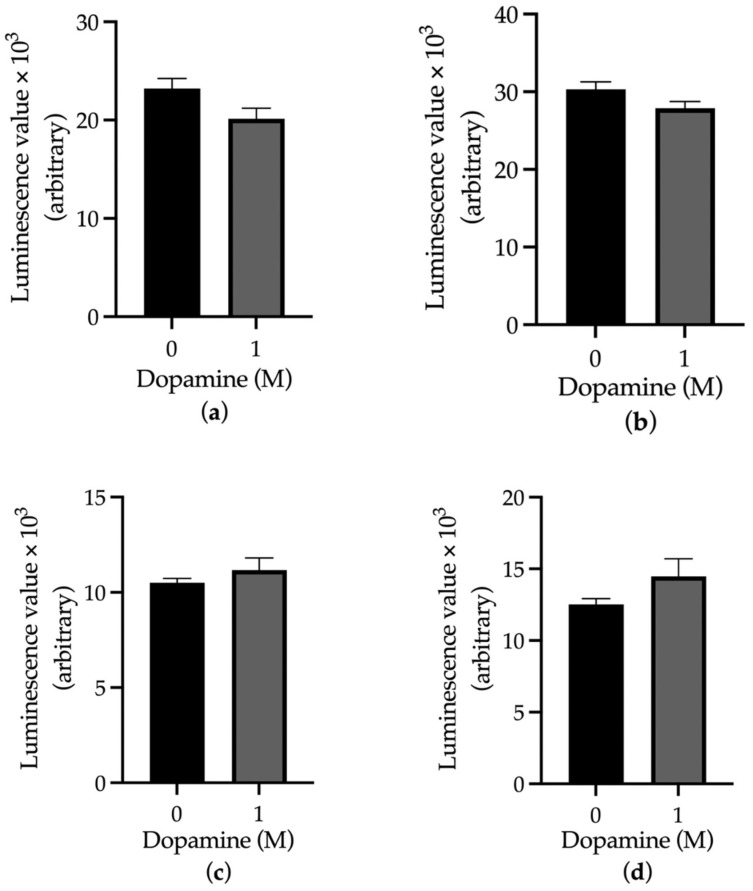
DRD4-Tango Reporter Analysis In vivo. DRD4-hTango reporter (luciferase) activity in response to dopamine in flies expressing: (**a**) NP1-Gal4 (the midgut driver) on the second chromosome, and both 8×LexAop and human DRD4 on the third chromosome (*n* = 6); (**b**) both NP1-Gal4 and human DRD4 on the second chromosome, and 8×LexAop on the third chromosome (*n* = 8); (**c**) NP1-Gal4 on the second chromosome, and both 13×LexAop and human DRD4 on the third chromosome (*n* = 4); and (**d**) both NP1-Gal4 and human DRD4 in the second chromosome, and 13XLexAop in the third chromosome (*n* = 3). Error bars represent the standard error of the mean (SEM), *n* = 3.

To investigate whether Tango reporter expression in *Drosophila* indicates the activation of human DRD4, we analyzed reporter expression following dopamine treatment. Administration of dopamine to DRD4-hTango flies for 6 h yielded no significant increase in reporter expression (Figure 5a–d), suggesting that the system was not functional in vivo.

## 3. Discussion

The successful validation of the DRD4-Tango system in *Drosophila* S2 cells is consistent with previous studies that have utilized the Tango assay in mammalian cells. The Tango system has previously been employed in mammalian cell lines, such as HEK293 cells, to study GPCR activity, where it has demonstrated high sensitivity and specificity for detecting receptor–ligand interactions [12,22]. Our current study extends this application to *Drosophila* S2 cells, demonstrating that the Tango system in these cells provides an adequate platform for studying human GPCRs and their ligands. These findings establish that *Drosophila* S2 cells represent a reliable model for human GPCR research, offering an alternative to mammalian systems with significant advantages including easier culture conditions and genetic manipulation.

The selectivity experiments, which demonstrated the system’s lack of response to octopamine (a non-ligand for DRD4), support the conclusion that the Tango system effectively discriminates between specific ligands and non-ligands. This selectivity aligns with previous findings showing the Tango assay’s ability to provide robust and specific readouts of GPCR activation [12]. These results demonstrate that *Drosophila* S2 cells, when engineered with the DRD4-Tango system, retain the necessary receptor–ligand specificity for GPCRs–ligand interaction studies. Such specificity is essential for future applications in high-throughput screening of potential therapeutic compounds.

The positive response of the DRD4-Tango system to a chromone-based compound demonstrates that this system is effective for studying known ligands and identifying novel GPCR-targeting compounds. This result validates the potential of the Tango system for drug screening applications. Previous studies have highlighted the importance of GPCRs as drug targets, with over one-third of currently marketed drugs acting on these receptors [23]. Our current study extends this body of knowledge by demonstrating that *Drosophila* S2 cells, combined with the Tango assay, provide an efficient platform for discovering new GPCR-targeting drugs, thereby accelerating the drug discovery process.

The challenges encountered in detecting DRD4 activation in vivo highlight potential limitations in the current approach, contrasting with the relatively straightforward results obtained in vitro. This discrepancy suggests that certain factors, such as low expression levels of the DRD4 receptor or suboptimal experimental conditions, may have contributed to the failure of the in vivo experiments. For instance, receptor expression in the in vivo system may have been insufficient to produce a detectable signal, which could be resolved by optimizing the transfection protocol or using stronger promoters to drive receptor expression. Additionally, the experimental conditions, including the duration of ligand exposure or temperature during the assay, may not have been optimal to support receptor–ligand interactions in vivo, necessitating further protocol adjustments. While previous studies have reported that translating in vitro findings to in vivo systems often requires significant optimization [22], our observations reinforce the importance of tailoring experimental designs for in vivo applications. Moving forward, efforts should focus on refining receptor expression strategies and optimizing assay conditions. These improvements are essential to realizing the full potential of the DRD4-Tango system as a robust tool for in vivo studies.

The findings of this study have broader implications for the use of *Drosophila* cells in GPCR research. By establishing that human GPCRs are functionally active in a *Drosophila* cell model, this research reveals new opportunities for exploring receptor pharmacology in a simpler and more genetically tractable system. Our results provide strong evidence supporting the potential of *Drosophila* S2 cells as a versatile platform in GPCR research. Future studies should focus on both optimizing in vivo applications and expanding the system to other GPCRs, potentially leading to significant advancements in both basic research and drug discovery.

## 4. Materials and Methods

### 4.1. Cell Culture, Plasmid Mixture Preparation, and Transfection

*Drosophila* S2 cells were maintained at 25 °C in Schneider’s *Drosophila* medium (Gibco, Thermo Fisher Scientific Inc., Waltham, MA, USA) in addition to 10% (*v*/*v*) heat-inactivated FBS, 100 units/mL penicillin, and 100 g/mL streptomycin. DNA encoding the full-length human β-arrestin1 with R393E/R395E double mutations (designated as ARRB1EE), which enhances GPCR binding [24], was gene synthesized by GenScript, as previously described [25]. DNA condensation buffer (Buffer EC) and an enhancer for the Effectene^®^ transfection reagent (#301425, QIAGEN, Hilden, Germany) were mixed with the desired plasmids; respectively, pMT-GAL4 (40 ng), UAS-DRD4-hTango (40 ng), and pJFRC-8xLexAop-NanoLuc (40 ng). The mixture was left at room temperature for five minutes before adding the Effectene^®^ transfection reagent (#301425, QIAGEN, Hilden, Germany) and incubated once more for five minutes at room temperature. S2 cells (1.5 × 10^6^ cells/mL) transfected with the plasmid mixture were incubated for 24 h at 25 °C. Copper sulfate (CuSO_4_) 1 mM was added to induce the metallothionein promoter of pMT-GAL4 and the cells were incubated at 28 °C for 24 h [26].

### 4.2. Plasmid Construction

#### 4.2.1. CB-Blunt-NanoLuc

We PCR amplified a NanoLuc fragment by using pUAS-NanoLuc (Addgene plasmid no. 87696) and a pair of primers (Nluc.F-XhoI-Kz: ctcgagcaaaATGGTCTTCACACTCGAAGATTTC and Nluc.R-XbaI; tctagaTTACGCCAGAATGCGTTCGCACAG). The amplified fragment was subcloned into a pCR-Blunt vector (Thermo Fisher Scientific Inc., Waltham, MA, USA) by ligation (Ligation High, TOYOBO Co., Ltd., Osaka, Japan) and the resultant clone was named pCB-Blunt-NanoLuc.

#### 4.2.2. pJFRC-8XLexAop2-NanoLuc

We digested the pCB-Blunt-NanoLuc and pJFRC18-8XLexAop2-mCD8::GFP (#26225, Addgene, Watertown, MA, USA) plasmids with XbaI and XhoI and ligased them (Ligation High) to construct a reporter plasmid pJFRC-8XLexAop2-NanoLuc.

#### 4.2.3. pJFRC-13XLexAop2-IVS-NanoLuc

For construction of another reporter plasmid, pJFRC-13XLexAop2-IVS-NanoLuc, we amplified a NanoLuc fragment by PCR with a pair of primers (Nluc.infF 5′-cggccgcggctcgagCAAAATGGTCTTCACACTCGAAG-3′ and Nluc.infR 5′-acaaagatcctctagATTACGCCAGAATGCGTTCGCAC-3′) and inserted it into pJFRC19-13XLexAop2-IVS-myr::GFP (#26224, Addgene, Watertown, MA, USA) linearized with XbaI and XhoI by using the In-fusion HD Cloning kit (TAKARA BIO Inc., Shiga, Japan).

#### 4.2.4. pUAS-DRD4-hTango

By using the Valium-TEVcs-LexA-HAtag-2A-Arrestin-TEVp [22], pCAGGS-ARRB1EE, and pEX-K4J1-Flag-V2 vectors (this study), we constructed a Valium-Kz-Flag-V2-TEVcs-LexA-3xHA-2A-ARRB1EE-TEVp-V5 plasmid, where Kz stands for a Kozak sequence (CAAC). The DRD4 fragment was PCR amplified from DRD4-Tango (#66271, Addgene, Watertown, MA, USA) and subcloned into a pCR-Blunt vector (pCR-Blunt-DRD4, Thermo Fisher Scientific Inc., Waltham, MA, USA). Then, a DRD4 fragment was amplified by PCR with a pair of primers, 5′-cagcatcgatcctagGATGGGTAATCGAAGCACTGCTGAC-3′ (DRD4-infF) and 5′-gggtgcgtcccttaaGACAGCAAGCCCGGAGAGCCTTC-3′ (DRD4-infR), and inserted into Valium-Kz-Flag-V2-TEVcs-LexA-3xHA-2A-ARRB1EE-TEVp-V5 digested with AflII and AvrII by In-fusion HD cloning (TAKARA BIO Inc., Shiga, Japan) to generate pUAS-DRD4-hTango.

### 4.3. Procedure for Synthesis of (E)-3-(4-methoxyphenyl)-1-(3-methyl-5-nitrophenyl)prop-2-en-1-one (***3***)

A 40% aqueous solution of KOH (15 mL) was added to a stirred solution of 2-hydroxy-5-methyl-3-nitrocetophenone (**1**, 10 mmol) and furfural (**2**, 10 mmol) in absolute methanol (30 mL). The mixture was sonicated for 1 h at 40 °C, followed by stirring at room temperature (rt) for 20 h. After the reaction was complete and monitored by thin-layer chromatography (TLC), the solution was acidified with 1 M HCl, resulting in the formation of a precipitate. The precipitate was filtered, rinsed with cold distilled water, and purified by flash chromatography using a gradient of 10–80% ethyl acetate in *n*-hexane. It was then subjected to recrystallisation using MeOH to afford crystal solids.

### 4.4. Procedure for Synthesis of 2-(Furan-2-yl)-3-hydroxy-6-methyl-8-nitro-4H-chromen-4-one (***4***)

A mixture of **3** (10 mmol), methanol (30 mL), potassium hydroxide (KOH, 40%, 15 mL), and hydrogen peroxide (H_2_O_2_, 30%, 10 mL) was stirred vigorously for 3 h at 0 °C to room temperature (rt). The reaction progress was monitored via TLC. Upon completion, the reaction mixture was poured into ice-cold water and acidified with 1 M HCl. The resulting precipitate was collected by filtration, washed with water, and recrystallized from ethanol.

### 4.5. Procedure for Synthesis of 2-(Furan-2-yl)-6-methyl-8-nitro-4-oxo-4H-chromen-3-yl Acetate (***5***)

**Scheme 1:**
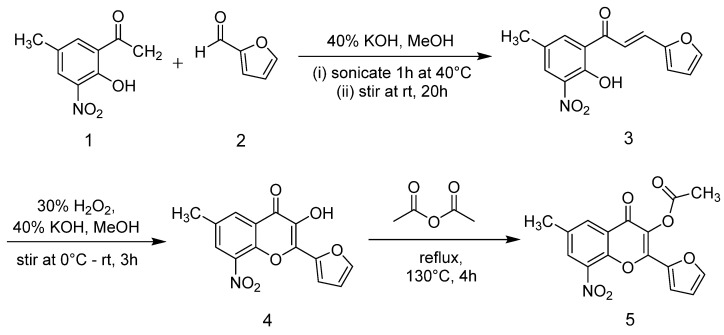
The synthetic route of 2-(furan-2-yl)-6-methyl-8-nitro-4-oxo-4*H*-chromen-3-yl acetate.

Compound **4** (4, 0.15 g, 0.5 mmol) was added to acetic anhydride (12 mL), and the mixture was heated to 130 °C for 4 h. After completion, the reaction mixture was poured onto ice and extracted with dichloromethane (DCM). The organic layer was washed with a saturated sodium bicarbonate (NaHCO_3_) solution, dried over anhydrous sodium sulfate (Na_2_SO_4_), filtered, and concentrated under reduced pressure. The crude product was then purified by column chromatography using a hexane-ethyl acetate solvent system and recrystallized using absolute EtOH to afford compound **4**. The product formed as a brownish crystal; yield 26%; m.p. 250–251 °C. ^1^H NMR (600 MHz, CDCl_3_) δ 8.32 (dd, J = 2.3, 1.0 Hz, 1H), 8.21 (d, J = 2.2 Hz, 1H), 7.77 (dd, J = 1.7, 0.8 Hz, 1H), 7.39 (dd, J = 3.6, 0.8 Hz, 1H), 6.80 (m, 1H), 6.68 (dd, J = 3.6, 1.7 Hz, 1H), 2.56 (s, 3H), 1.56 (s, 3H). 13C NMR (151 MHz, CDCl3) δ 170.90, 145.89, 145.29, 143.91, 139.29, 136.17, 134.43, 130.84, 130.74, 122.82, 116.58, 112.74, 59.47, 38.09, 31.17, 20.71. The scheme of the synthetic route of 2-(furan-2-yl)-6-methyl-8-nitro-4-oxo-4*H*-chromen-3-yl acetate is described in the Scheme 1.

### 4.6. In Vitro DRD4-hTango Assay

Independently, 0.1 M Dopamine hydrochloride (#14212-84, Nacalai Tesque, Kyoto, Japan), 0.1 M Octopamine hydrochloride, and 20 μM of 2-(furan-2-yl)-6-methyl-8-nitro-4-oxo-4*H*-chromen-3-yl acetate were added to the cells as samples and left in 28 °C for one hour. Luminescent signals from the cells were measured using Nano-Glo^®^ Luciferase Assay System (#N1120, Promega, Madison, WI, USA).

### 4.7. Fly Stock

We injected pJFRC-8XLexAop2-NanoLuc and pJFRC-13XLexAop2-IVS-NanoLuc plasmid DNA into y [1] M{vas-int.Dm}ZH-2A w[*]; PBac{y[+]-attP-3B}VK00033 (KYOTO *Drosophila* Stock Center, DGRC # 130448) embryos and established *y [2] cho [2] v [1]; PBac{y[+mDint2] v[+t1.8] = 8xLexAop2-hsp-Nluc.1}VK00033* and *y [2] cho [2] v [1]; PBac{y[+mDint2] v[+t1.8] = 13xLexAop2-hsp-Nluc.2}VK00033* strains, respectively. pUAS-DRD4-hTango plasmid DNA was injected into *y [1] v [1] P{y[+t7.7] = nos-phiC31\int.NLS}X; P{y[+t7.7] = CaryP}attP40* (Bloomington *Drosophila* Stock Center, BDSC, #25709) and *y [1] sc [1] v [1] P{y[+t7.7] = nos-phiC31\int.NLS}X; P{y[+t7.7] = CaryP}attP2* (BDSC # 25710) embryos to construct *y [2] cho [2] v [1]; P{y[+t7.7] v[+t1.8] = UAS-hsp-Hsap\DRD4.hTango.3}attP40* and *y [2] cho [2] v [1]; P{y[+t7.7] v[+t1.8] = UAS-hsp-Hsap\DRD4.hTango.3}attP* strains.

Four strains of Drosophila melanogaster, respectively, y [2] cho [2] v [1]; P{y+t7.7 v+t1.8=UAS-Hsap-DRD4-hTango.3}attP2 (hereby UAS-DRD4-hTango_attP2), y [2] cho [2] v [1]; P{y+t7.7 v+t1.8=UAS-Hsap-DRD4-hTango.3}attP40 (hereby {UAS-DRD4-hTango}attP40), NP1-GAL4, tub-Gal80ts/CyO; {8×lexAop2_Nluc}VK0003/TM6B (hereby NP1-8×), and NP1-GAL4, tub-Gal80ts/CyO; {13×exAop2_Nluc}VK0003/TM6B (hereby NP1-13×), were reared inside glass vial containing cornmeal agar with composition of 4% cornmeal powder, 8% dried brewer’s yeast powder, and 10% glucose, with butyl p-hydroxybenzoate and propionic acid as preservatives. UAS-DRD4-hTango strains were received from Dr. Kotaro. Adult male UAS-DRD4-hTango_attP2, adult male {UAS-DRD4-hTango}attP40, adult virgin NP1-8×, and adult virgin NP1-13× were separately crossed together based on Supplementary Figure S1. The parents were discarded, and hatched transgenic larvae were kept at 25 °C until the pupal stage. The vials containing progeny pupae were then moved and kept at 18 °C until eclosion.

### 4.8. In Vivo DRD4-hTango Assay

In vivo DRD4-hTango assay as per Chen et al., 2020, with modifications [27]. The newly hatched female progenies were collected and transferred into vials containing 2.5% sucrose and 1.5% agar and kept at 18 °C for three days. Vials were then incubated in 29 °C for 14 h to activate the Tango system. Starvation was performed for two hours before flies were continuously fed with 1 M dopamine hydrochloride in water (1% natrium bisulphite was added into the mix to inhibit the oxidation process of dopamine) for six hours. Food was replaced every 2 h to ensure the dopamine intake.

Midgut samples were retrieved by dissecting the flies. The samples were collected inside a 1.5 mL BioMasher disposable homogenizer tube (#893062, Nippi Inc., Tokyo, Japan) containing NB buffer (150 mM NaCl, 50 mM Tris-HCl pH 7.5, 2 mM EDTA, 0.2% NP-40 as per [27], with modifications [27], and a protease inhibitor cocktail with 1x final concentration to inhibit protein degradation. Samples were homogenized and mixed by tapping every five minutes for 30 min on ice. Samples were centrifuged (16,000× *g*) for 10 min and the luminescence signal of the collected supernatant was then measured using the Nano-Glo^®^ Luciferase Assay System (Promega, Madison, WI, USA).

### 4.9. Statistical Analysis

All data are displayed as the mean ± standard error mean (SEM). The data were tested for normality using Shapiro–Wilk normality test. Data that satisfy normality are further analyzed by one-way analysis of variance ANOVA (Tukey post test) or t-test to determine whether differences existed across treatment groups using GraphPad Prism 10 software. *p*-values of < 0.05 were deemed statistically significant.

## Data Availability

The original contributions presented in this study are included in the article/Supplementary Materials. Further inquiries can be directed to the corresponding author(s).

## Supplementary Materials

The following supporting information can be downloaded at: https://www.mdpi.com/article/10.3390/ijms26010202/s1.
