# Supplementary material for: Detection of Human GPCR Activity in Drosophila S2 Cells Using the Tango System
Source: Int J Mol Sci. 2024 Dec 29;26(1):202. doi: 10.3390/ijms26010202 (PMC11720185; doi:10.3390/ijms26010202)
Supplement: Supplementary file 1 [file ijms-26-00202-s001.zip › ijms-3378215-supplementary.pdf]

Detection of Human GPCR Activity in Drosophila S2 Cells Using the Tango System

Emil Salim [1,2*], Aki Hori [1], Kohei Matsubara [3], Toshiyuki Takano-Shimizu [3], Andre Pratomo [1], Marianne [2], Armia Syahputra [4], Dadang Irfan Husori [2], Asuka Inoue [5, 6], Maryam Aisyah Abdullah [7], Nur Farisya Shamsudin [7], Kamal Rullah [7], and Takayuki Kuraishi [1*]

Supplementary figure

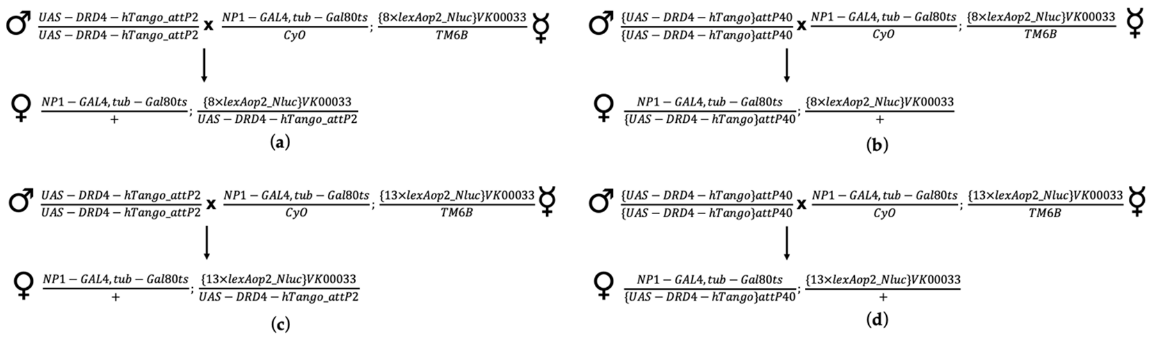

Supplementary Figure S1. Mating Schemes of In Vivo Tango Assay for *Drosophila melanogaster*. **(a)** Generation of transgenic flies expressing NP1-Gal4 (the midgut driver) on the second chromosome, and both 8×LexAop and the human DRD4 in the third chromosome, **(b)** both NP1-Gal4 and the human DRD4 in the second chromosome, and 8×LexAop on the third chromosome, **(c)** NP1-Gal4 on the second chromosome, and both 13×LexAop and the human DRD4 on the third chromosome, **(d)** both NP1-Gal4 and human DRD4 on the second chromosome, and 13×LexAop on the third chromosome.
